# Preparation and Characterization of Surface Heat Sintered Nanohydroxyapatite and Nanowhitlockite Embedded Poly (Lactic-co-glycolic Acid) Microsphere Bone Graft Scaffolds: In Vitro and in Vivo Studies

**DOI:** 10.3390/ijms21020528

**Published:** 2020-01-14

**Authors:** Gils Jose, K.T. Shalumon, Han-Tsung Liao, Chang-Yi Kuo, Jyh-Ping Chen

**Affiliations:** 1 Department of Chemical and Materials Engineering, Chang Gung University, Kwei-San, Taoyuan 33302, Taiwan; 2 Department of Plastic and Reconstructive Surgery and Craniofacial Research Center, Chang Gung Memorial Hospital, Linkou, Chang Gung University School of Medicine, Kwei-San, Taoyuan 33305, Taiwan; 3 Research Center for Food and Cosmetic Safety, Research Center for Chinese Herbal Medicine, College of Human Ecology, Chang Gung University of Science and Technology, Taoyuan 33302, Taiwan; 4 Department of Materials Engineering, Ming Chi University of Technology, Tai-Shan, New Taipei City 24301, Taiwan

**Keywords:** whitlockite, hydroxyapatite, poly (lactic-co-glycolic acid), microsphere, bone graft, bone marrow stem cells

## Abstract

In the context of using bone graft materials to restore and improve the function of damaged bone tissues, macroporous biodegradable composite bone graft scaffolds have osteoinductive properties that allow them to provide a suitable environment for bone regeneration. Hydroxyapatite (HAP) and whitlockite (WLKT) are the two major components of hard tissues such as bone and teeth. Because of their biocompatibility and osteoinductivity, we synthesized HAP (nHAP) and WLKT nanoparticles (nWLKT) by using the chemical precipitation method. The nanoparticles were separately incorporated within poly (lactic-co-glycolic acid) (PLGA) microspheres. Following this, the composite microspheres were converted to macroporous bone grafts with sufficient mechanical strength in pin or screw shape through surface sintering. We characterized physico-chemical and mechanical properties of the nanoparticles and composites. The biocompatibility of the grafts was further tested through in vitro cell adhesion and proliferation studies using rabbit bone marrow stem cells. The ability to promote osteogenic differentiation was tested through alkaline phosphate activity and immunofluorescence staining of bone marker proteins. For in vivo study, the bone pins were implanted in tibia bone defects in rabbits to compare the bone regeneration ability though H&E, Masson’s trichrome and immunohistochemical staining. The results revealed similar physico-chemical characteristics and cellular response of PLGA/nHAP and PLGA/nWLKT scaffolds but the latter is associated with higher osteogenic potential towards BMSCs, pointing out the possibility to use this ceramic nanoparticle to prepare a sintered composite microsphere scaffold for potential bone grafts and tissue engineered implants.

## 1. Introduction

Bone fractures and bone disorders among middle aged people are increasing day by day, which demands the development of suitable bone grafts as an essential prerequisite to fix bone defects. Recent advancements in bone graft materials including polylactide, titanium, and hydroxyapatite (HA) have led to the introduction of various products including bone plates and biodegradable bone pins that can cure many bone defects [1,2]. Although many bone grafts and similar tissue engineering scaffolds possess biodegradability, biocompatibility, and adequate mechanical strength, their ability to reconstruct defected areas has not been very high in many experiments [3]. Hence, developing a bio-absorbable bone graft with osteogenic properties will be helpful to achieve a better success rate. Osteobiologic materials are engineered biomaterials that can serve as implant materials for bone repair and remodeling. Due to the capacity of these osteobiologic materials to promote the healing of bone fractures, they have been the most discussed biomaterials in the past decade [4]. The osteobiologic materials are expected to provide a biocompatible surface that can promote cell attachment and migration after implantation. Due to the similarities of these materials with the body, the scaffolds made of osteobiologic materials can mimic the bodies’ inherent capacity to heal defects and to regenerate bone tissues [5]. Although various organic materials have been used in clinical applications for bone regeneration, using bioceramics to treat damaged hard tissue has also gained significant attention [6]. Among the biomimicking ceramics, calcium phosphate-based bioceramics have been widely used due to their compositional similarity with human hard tissue and excellent biocompatibilities [7]. Hydroxyapatite (Ca_10_(PO_4_)_6_(OH)_2_) and whitlockite (Ca_18_Mg_2_(HPO_4_)_2_(PO_4_)_12_) are the most widely used materials in this family due to their osteoconductivity and their compositional and crystallographic similarities with natural bone materials [8]. Moreover, nanoparticles prepared from these bioceramic materials can further enhance their physical as well as biological properties, due to the greater surface area provided [9,10].

Hydroxyapatite (HAP), the most commonly used form of calcium phosphate ceramics, has been used for a variety of biomedical applications, including matrices for controlled drug release and bone tissue engineering scaffolding materials [11,12]. Due to the chemical similarity of HAP with the inorganic component of a bone matrix, it exhibits osteoconductivity as well as a strong affinity towards hosting hard tissues. It possesses high thermodynamic stability in physiological conditions and is able to form direct chemical bonding to the bone [13]. In addition to this, synthetic HAP offers high biocompatibility, slow biodegradability in situ, as well as good osteoconductive and osteoinductive properties [14,15]. On the other hand, whitlockite (WLKT), consisting of Ca^2+^, Mg^2+^, and PO_4_^3−^ ions, is another component that constitutes about 20 to 30 wt% of the inorganic phase of human bone [16,17]. The WLKT is the second most abundant ceramic material present in bones and also known to exist with a high ratio in early stage bone tissue formation [18,19]. As bone stores 99% of the Ca^2+^ and more than 50% of the Mg^2+^ in our body, WLKT is used as a storehouse for Mg^2+^ since the other major bone mineral HAP could hardly accommodate Mg^2+^ in its lattice structure [19]. Although biocompatibility and chemical compositions of WLKT and HAP are quite similar, the capacity of WLKT to enhance both cell proliferation and mineralization is comparatively high compared to HAP [20]. In addition to this, different studies suggest the stability of WLKT in acidic condition is higher than HAP [21]. The acidic stability of WLKT plays a major role in its capability to promote bone mineralization. This property is connected with the better resorption of organic bone matrices through proteinase in an acidic pH condition, which is necessary for bone formation. Even though HAP and WLKT hold all the biological properties required for rapid bone formation, the low mechanical strength and fracture toughness act as an obstacle for the load-bearing applications [22]. Hence, the fabrication of composites by incorporating bioceramics in polymeric materials is an obvious solution for the aforementioned problem [14]. Among the various polymeric materials available, poly (lactic-co-glycolic acid) (PLGA) is a biocompatible and biodegradable material that can provide enough mechanical strength for the composite to act as a bone graft [23].

**Figure 1:**
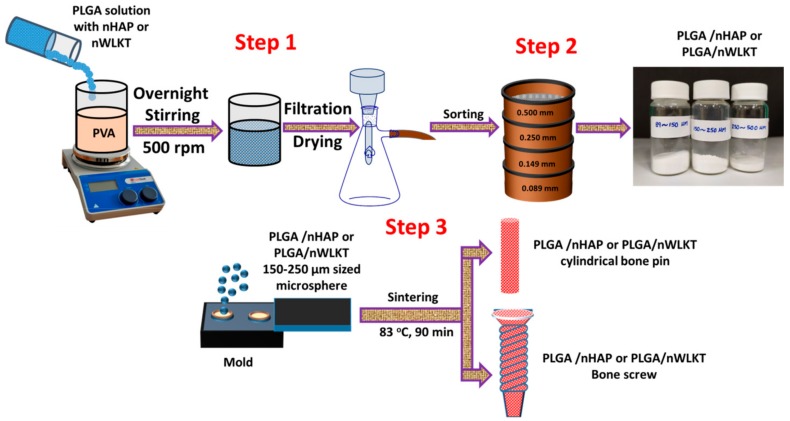
The schematic representation of poly (lactic-co-glycolic acid) (PLGA)/nHAP or PLGA/nWLKT microsphere preparation (step 1, 2) and the subsequent fabrication process of microsphere scaffold by using surface sintering (step 3).

In this work, we prepared a composite PLGA microsphere containing nanosized HAP (nHAP) or WLKT (nWLKT) to combine the advantages of improved mechanical properties of a polymeric material and the osteogenic properties of bioceramic nanoparticles (Figure 1, step 1 and 2). The composite microspheres were heat sintered to fabricate macroporous bone grafts in different shapes such as pins and screws (Figure 1, step 3). The PLGA/nHAP and PLGA/nWLKT microsphere based bone grafts thus fabricated were subsequently used for comparison of the physico-chemical, mechanical properties, and biological properties. In vitro studies were then conducted using rabbit bone marrow stem cells (BMSCs) to study cell attachment, proliferation, and differentiation within the porous grafts. Further, in vivo experiments were performed using the rabbit tibia defect model to examine the efficacy of the grafts for bone regeneration.

## 2. Results and Discussion

### 2.1. Characterization of nHAP and nWLKT

**Figure 2:**
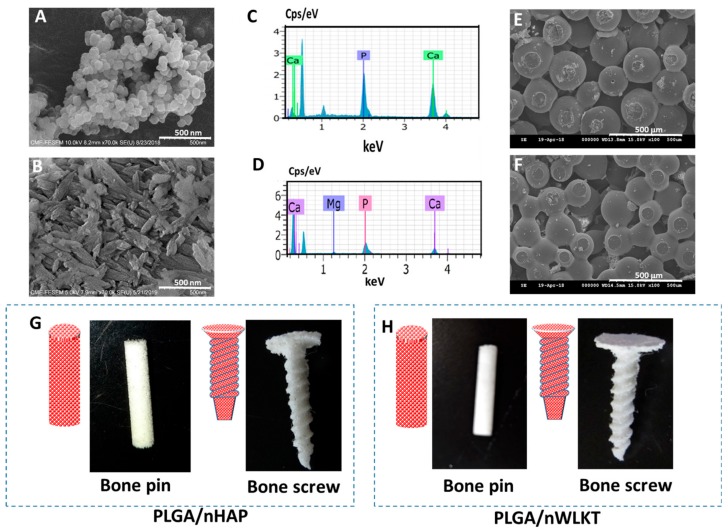
The characterization of nHAP and nWLKT by using scanning electron microscopy (SEM) (**A**,**B**) and electron dispersive X-ray spectroscopy (EDS) (**C**,**D**). The SEM images of PLGA/nHAP microsphere scaffold (**E**) and PLGA/nWLKT microsphere scaffold (**F**) confirm uniform surface sintering. Schematic drawings and photographs of PLGA/nHAP (**G**) and PLGA/nWLKT (**H**) bone grafts in a pin and screw shape after the surface sintering process.

The morphology of prepared nHAP and nWLKT particles was analyzed using scanning electron microscopy (SEM) analysis, from which particles were found to be uniform in size and shape, and with minimum agglomeration (Figure 2A,B). The nHAP particles displayed a round-shaped morphology with an average diameter of ~100 nm (Figure 2A), while nWLKT particles showed its characteristic rhombohedral shape (Figure 2B) [16]. The elemental compositions of both samples were identified through energy dispersive X-ray spectroscopy (EDS) analysis. In nHAP, the measured calcium (Ca) to phosphate (P) stoichiometric ratio was 1.87 (Figure 2C). As this value is slightly higher than the theoretical value of 1.67, we hypothesized that this might be due to the formation of calcium-rich hydroxyapatite. The EDS spectrum of nWKLT revealed the presence of Mg elemental peak with its Ca_18_Mg_2_(HPO_4_)_2_(PO_4_)_12_ molecular structure in contrast to Ca_10_(PO_4_)_6_(OH)_2_ for nHAP (Figure 2D). Moreover, the experimental stoichiometric ratio of Ca/P was 1.26, close to the theoretical value of 1.29.

### 2.2. Characterization of PLGA/nHAP and PLGA/nWLKT

PLGA microspheres containing nHAP or nWLKT with sizes ranging from 150 to 250 µm were chosen from the crude samples and further fabricated into scaffolds in pin or screw shapes (Figure S1 (Supplementary Materials)). The SEM images of the microspheres are shown in Figure S1. In order to obtain the required microsphere size for surface sintering, the PLGA concentration and rotation speed during the mixing process were optimized to be 13.3% and 600 rpm. The PLGA concentration and rotation speed were optimized by using the trial-and-error method. As the concentration varied, the size and size distribution of the microspheres as well as the yield of microspheres changed (Figure S2, Supplementary Materials). All microspheres were washed in double distilled (DD) water to avoid surface attachment of PVA and were dried prior to storage in a desiccator. Since it is desirable to reach similar final concentrations of nHAP and nWLKT in the microsphere, an initial trial-and-error pilot study was conducted to estimate the final loading percentage of nHAP and nWLKT in the microsphere beforehand. It was found that the encapsulation efficacy of nHAP and nWLKT in the composite microspheres was drastically different, as the latter showed a lower value. A series of experiments with a range of nWLKT concentrations in PLGA solution further concluded that encapsulation of 1.2 g nWLKT in 4 g PLGA led to the same encapsulation amount of nanoparticles using 0.8 g nHAP and 4 g PLGA. This was confirmed by the thermogravimetric analysis (TGA) shown in Figure S3 (Supplementary Materials). This condition was thus utilized for preparing PLGA/nHAP and PLAG/nWLKT microspheres. The SEM images of sintered microsphere scaffold of PLGA/nHAP (Figure 2E) and PLGA/nWLKT (Figure 2F) confirmed the uniform surface-to-surface sintering with an average pore size of 234 ± 64 µm and 286 ± 82 µm, which could be seen in the lower and higher magnification SEM images in Figure S4 (Supplementary Materials). The gross views of the pin and screw shaped scaffolds of PLGA/nHAP (Figure 2G) and PLGA/nWLKT (Figure 2H) indicate that the intended shape and morphology of the bone graft could be achieved through surface sintering of the composite microspheres, which endorsed the possibility of applying this manufacturing process to fabricate bone grafts with suitable shapes for bone surgeries. Another relevant issue while considering scaffolds for tissue replacements is the porosity. Although our bone pin was intended as an acellular bone graft implant without cells, its porosity was also crucial for cellular penetration of stem cells from the surrounding tissues into the graft interior. It therefore was helpful to encompass the use of a 3D scaffold to provide a suitable microenvironment for cell ingrowth and differentiation to regenerate damaged tissues [24]. Since macroporous materials can also efficiently handle waste and nutrient exchange more efficiently during the regeneration phase, achieving adequate porosity without tailoring mechanical properties is a tactical factor in bone graft development. The fabricated bone pin developed here attained a porosity of 49.6% (PLGA/nHAP) and 47.3% (PLGA/nWLKT), which is sufficient for cell penetration [25].

**Figure 3:**
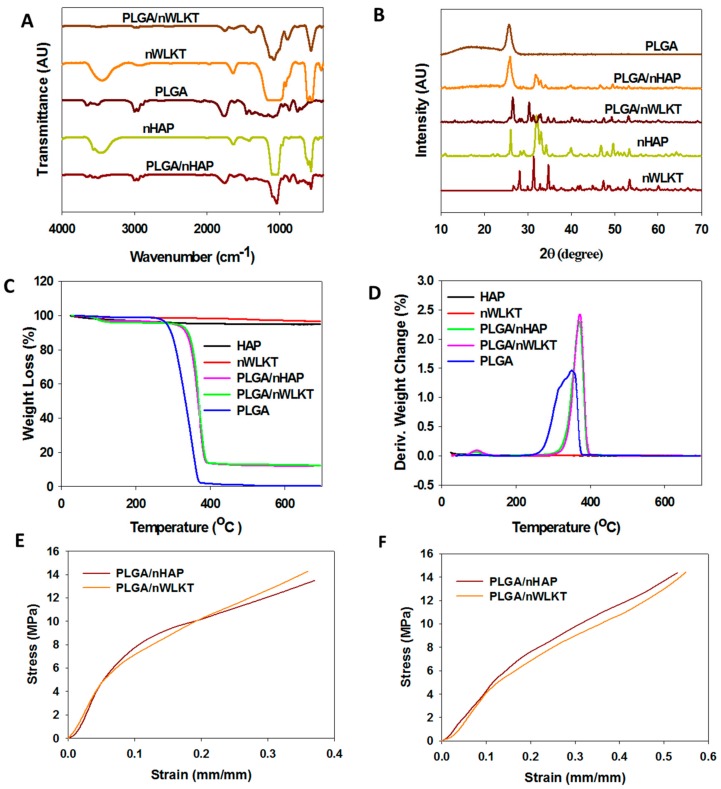
Characterization of nHAP, nWLKT, PLGA, PLGA/nHAP, and PLGA/nWLKT by Fourier-transformed infrared spectroscopy (FTIR) (**A**), X-ray diffraction (XRD) (**B**), thermogravimetric analysis (TGA) (**C**), and differential thermogravimetry (DTG) (**D**). The stress-strain curves of PLGA/nHAP and PLGA/nWLKT microsphere scaffold obtained through compressive mechanical testing are shown in (**E**) and (**F**) for dry and wet samples.

The Fourier-transformed infrared spectroscopy (FTIR) analysis was conducted to identify the chemical compositions within the microsphere scaffolds (Figure 3A). The characteristic stretching and bending vibrations of the scaffolds were recorded and compared with the pristine nHAP, nWLKT and PLGA. Considering the nHAP spectrum, phosphate stretching vibrations were observed at 564, 617, and 1030 cm^−1^. The band at 1412 cm^−1^ belonged to the hydroxyl group. The sharp peak at 1644 cm^−1^ and a broad absorption at 3339–3579 cm^−1^ might be due to the moisture present in the samples. nWLKT also had similar peaks due to its structural resemblance with nHAP where bands at 542 and 617 cm^−1^ could be assigned to the phosphate group, while bands at 3444 cm^−1^ could be due to the hydroxyl stretching. PLGA displayed characteristic peaks for the alkyl group in the broad range of 2942–3010 cm^−1^, the hydroxyl vibrations were observed at 760 and 3526–3669 cm^−1^, C=O stretching at 1765cm^−1^ and C-C stretching bands at 1104 cm^−1^ [26]. Both PLGA/nHAP and PLGA/nWLKT microsphere scaffolds displayed all characteristic peaks associated with PLGA, nHAP, and nWLKT. The bands at 572, 617, 1037, 1435, 1637, and 3514 cm^−1^ as well as at 580, 1089, 1644, and 3022 cm^−1^ denoted the presence of nHAP and nWLKT, respectively. Similarly, the peaks that appeared around 767, 872, 1773, and 3010 cm^−1^ in both scaffolds confirmed the presence of PLGA. Thus, the FTIR study clearly proved the effective incorporation of nHAP and nWLKT in PLGA microsphere scaffolds. In addition to FTIR, the presence of the ceramic counterparts in the composites was further examined through the identification of crystalline peaks by X-ray diffraction (XRD) analysis. As shown in Figure 3B, nHAP had major diffraction peaks at 2θ values 25.9 and 32°, along with other minor peaks at 33.1, 34.2, 39.9, 46.8, and 49.5°. PLGA had only a single diffraction peak at 25.5°. When combined with nHAP, PLGA/nHAP displayed all the characteristic peaks associated with nHAP and PLGA (Figure 3B). When examining the XRD pattern of nWLKT, peaks at 2θ values of 26.5, 31.2, and 34.7° were found to be unique in comparison with the nHAP pattern, which again all appeared in the pattern of PLGA/nWLKT.

The thermal stability of all samples was evaluated using thermogravimetric analysis (TGA). As shown in Figure 3C, PLGA copolymer possesses low thermal stability compared with ceramics and started to decompose at 180 °C, while complete decomposition occurred at around 340 °C [27]. In contrast, nHAP and nWLKT remained almost unchanged in weight up to 700 °C. The TGA curves for both PLGA/nHAP and PLGA/nWKLT showed ~12.4% residual weight when the temperature was increased to 700 °C, indicating the same weight ratio of the ceramic material (12.4%) was loaded into the composite microspheres. As explained before, it was observed that the loading efficiency of nWLKT in the microspheres were lower than that of nHAP during the emulsification step and therefore pilot TGA experiments were performed with different nWLKT loading concentrations to achieve a desirable final concentration of nWLKT in the microspheres. The overlapping TGA curves PLGA/nHAP and PLGA/nWLKT therefore justified the rationale to compare two different composite scaffolds based on the same ceramic content in the bone graft implant. The higher thermal stability of PLGA/nHAP or PLGA/nWLKT could be further confirmed from the increase of peak decomposition temperature from 346 °C to 375 °C after incorporating nHAP or nWKLT in PLGA (Figure 3D).

As a bone pin scaffold was intended to be applied in a load bearing condition, the compressive mechanical properties of the scaffolds were examined first. The respective stress-strain curves of PLGA/nHAP and PLGA/nWLKT bone pins in a dry state are shown in Figure 3E. The Young’s modulus and the yield stress were found to be similar for both pin-shaped scaffolds, which showed good mechanical properties with unbreakable strength, and even reached the maximum loading capacity of the testing machine. The Young’s modulus calculated from the stress-strain curve was 80.39 ± 17.5 for PLGA/nHAP and 77.25 ± 13.1 MPa for PLGA/nWLKT, which is of suitable mechanical strength to be used as a bone substitute. The high mechanical strength of both scaffolds could be ascribed to their surface-to-surface interconnectivity, which offers physical surface sintering and blending of surface PLGA in neighboring microspheres after heating. In order to evaluate the mechanical properties that simulate the wet condition in vivo, the samples were immersed in PBS for one hour before the mechanical testing. As shown in Figure 3F, the stress-strain curves which showed a similar trend to before were obtained in the wet state, where scaffolds remained unbroken, even after reaching the maximum loading capacity. Nonetheless, the Young’s modulus decreased to 37.37 ± 8.7 (PLGA/nHAP) and 39.86 ± 11.8 MPa (PLGA/nWLKT) and no significant difference was found to in the dry state.

### 2.3. In Vitro Studies

#### 2.3.1. Cell Morphology

**Figure 4:**
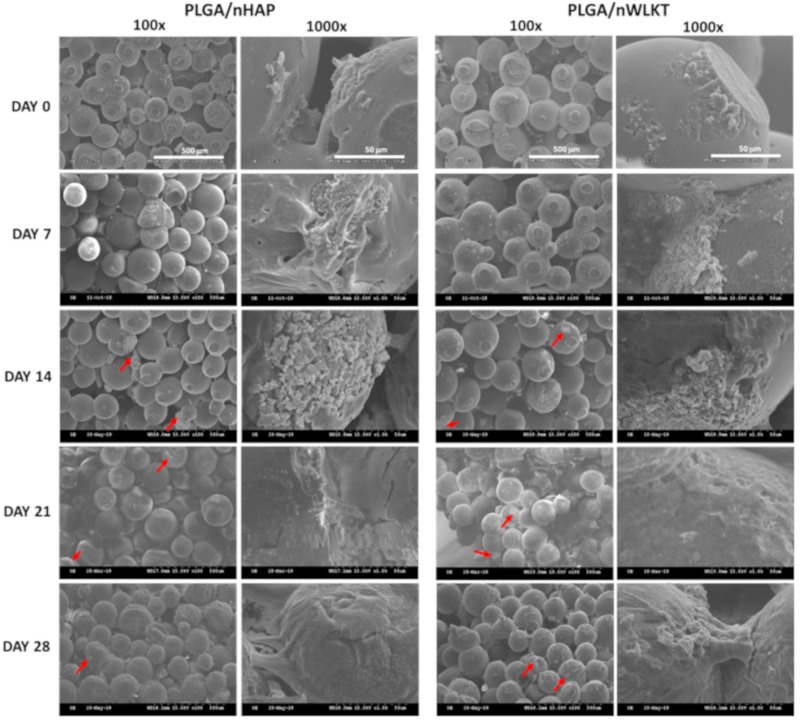
Assessment of cell morphology in PLGA/nHAP and PLGA/nWKLT microsphere scaffolds on day 0, 7, 14, 21, and 28 through scanning electron microscopy (SEM) at 100× (bar = 500 μm) and 1000× (bar = 50 μm) magnification. The red arrows indicate mineralized nodules.

The morphology of BMSCs cultured in PLGA/nHAP and PLGA/nWLKT microsphere scaffolds up to 28 days was observed under SEM and reported in Figure 4. Though the graft is an acellular implant scaffold, possible cell interaction after in vivo grafting should be assessed through cellular response from in vitro BMSCs culture. At day 0, the cells displayed more rounded morphology in both samples by weakly adhering to the microsphere surface. However, a rapid change in adhesion pattern was observed after seven days, where most of the cells spread alongside the curved surface of the microsphere. A higher density of cell population was observed within the pores of the scaffold on day 14, confirming the presence of homogenous cell growth that was ideal for regeneration. Cells started to migrate to the whole microsphere surface and also to the microsphere intersections after 21 days, leading to bridging between microsphere surfaces while filling the pores, which is ideal for tissue regeneration [28]. A thick cell covering layer was found on day 28 for both scaffolds. Apart from covering individual microspheres, the pores inside the scaffolds were also filled with cellular extensions due to continued cell growth with time. The mineralized nodules observed after 14 days further confirmed the initiation of osteogenic differentiation of BMSCs towards the bone lineage. More interestingly, there was no significant difference in cell spreading morphology between scaffolds, which confirmed the identical cellular responses of nHAP and nWLKT towards cell adhesion.

**Figure 5:**
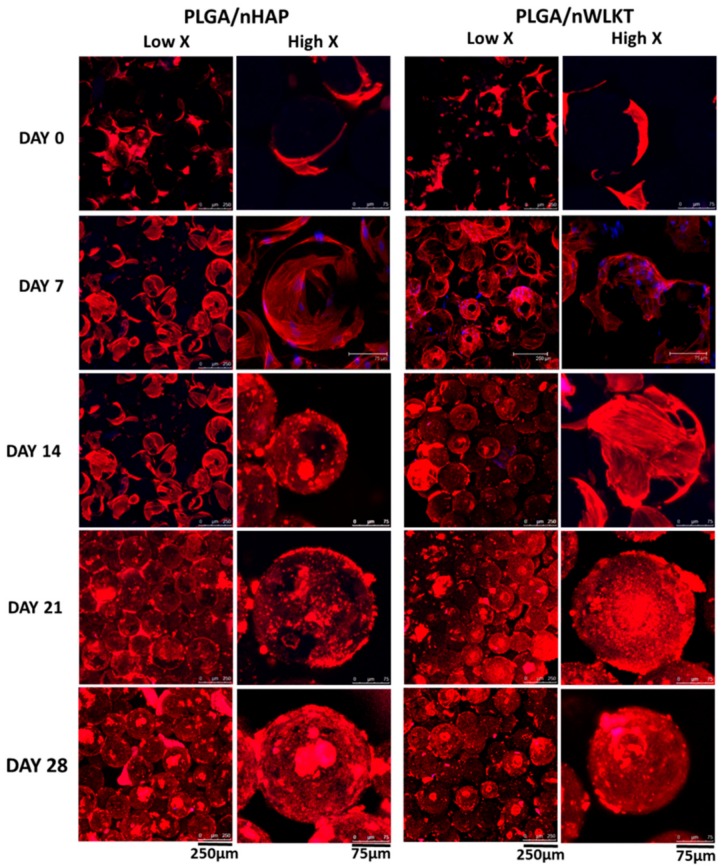
Visualization of the cytoskeletal arrangement in PLGA/nHAP and PLGA/nWLKT scaffolds through F-actin staining at low and high magnifications. Cell cytoskeleton was labeled with red fluorescence-producing phalloidin-tetramethylrhodamine B isothiocyanate and cell nuclei were counterstained with blue fluorescence-producing Hoechst 33,342 (Bar: low X = 250 µm, high X = 75 µm).

The cell morphology was further validated through staining of the actin cytoskeleton of adhered cells [29]. It is well known that the cytoskeleton has a vital role in determining the morphology of cells, the pattern of adhesion and the subsequent cell growth through signaling [30], and therefore it is crucial in cell migration and division. The major components of cytoskeleton are actin filaments, intermediate filaments, and microtubules with F-actin as the backbone. Unlike SEM analysis, actin staining helped to visualize the migration and proliferation of the cells within the three-dimensional structure of the scaffold, due to the versatility of the confocal microscope to scan various planes while providing a maximum projection. As observed from SEM analysis, the cells shifted from a more rounded and less intense cell adhesion pattern to a well spread and high intense adhesion scenario from day 0 to day 28 (Figure 5). Consistent with the SEM observation, less F-actin expression was detected due to early cell adhesion on day 0. A monolayer of more spread cell morphology on the microsphere was found after seven days and the pattern of cell spreading in both scaffolds on day 14 was slightly different from early stages due to possible cell differentiation. A preferential cell growth around the gap within the microsphere intersections was found at later stages. The relatively brighter spots in the scaffolds at these stages could be correlated with the multiple layering of cellular extensions, as clearly visible from the higher magnification images of individual microspheres. Thus, the cell morphology analysis confirmed the curved cell-spreading around the microspheres with an enhancement of F-actin stretching with respect to time. Interestingly, both scaffolds exhibited similar cellular cytoskeleton arrangements, with similar F-actin stress fibers within the gaps of microspheres. More detailed studies could reveal the relative efficiency of PLGA/nHAP and PLGA/nWLKT scaffolds towards bone regeneration from a bone graft aspect.

#### 2.3.2. Cell Viability and Proliferation

**Figure 6:**
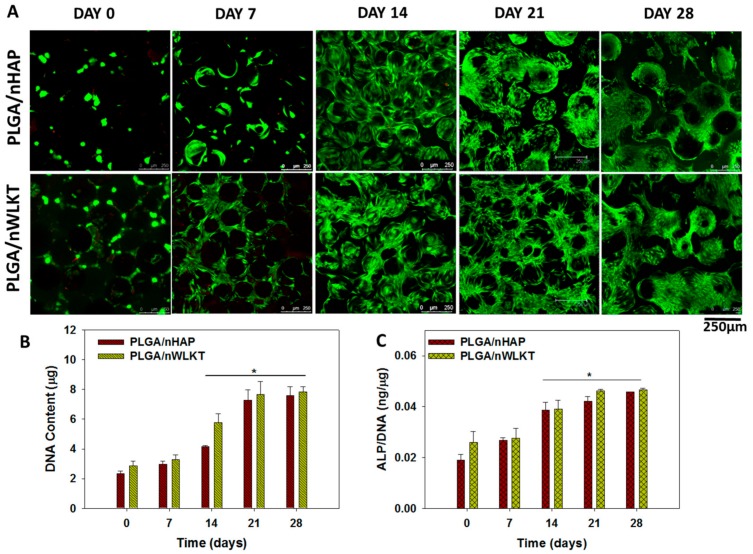
(**A**) Cell viability assessment of BMSCs in PLGA/nHAP and PLGA/nWLKT microsphere scaffolds through Live/Dead staining (bar = 250 µm). The quantitative estimation of cell proliferation and osteogenic differentiation of BMSCs seeded in the microsphere scaffolds was determined from DNA content (**B**) and normalized ALP activity (**C**) (* *p* < 0.05 compared to day 0).

The biocompatibility of both scaffolds was evaluated through the Live/Dead cell viability staining displayed in Figure 6A. The results obtained for the Live/Dead staining show a similar trend as observed in Figure 4 and Figure 5. Furthermore, the percentage of dead cell (red) in both scaffolds throughout the observation period up to 28 days was negligible in comparison with viable cells (green), and thus confirms the excellent biocompatibility of the scaffolds. As seen in the cell morphology pattern earlier, early stage cell adhesion resulted in less cell density and rounded morphology, while later stages revealed more spread cells. Only scattered live cells were visualized as green spots on day 0 during the initiation of cell spreading after attachment, which developed into a more cell-covered microsphere during cell proliferation to result in an ascending order of green fluorescence intensity with time. Similar to previous results, both scaffolds showed similar cellular response results with no distinguishable differences from Live/Dead staining.

Quantitative estimation of cell proliferation is essential to investigate the trend of increasing cell density around microspheres seen earlier. The rationale behind selecting BMSCs is due to its better proliferation and differentiation capabilities in a 3D micro-environment to mimic the natural architecture in bone [31]. Therefore, BMSCs cultured in both scaffolds were analyzed for cell proliferation through DNA analysis (Figure 6B). The DNA content increased with time due to cell division, which re-confirmed the results from SEM (Figure 4), the cytoskeleton expression from F-actin staining (Figure 5), and the Live/Dead cell viability assay (Figure 6A). The DNA content was lowest on day 0, increased before reaching a maximum at 21 days and plateaued thereafter. There was no significant difference in DNA content between PLGA/nHAP and PLGA/nWLKT throughout the culture period. The appearance of a cell number plateau during cell proliferation should be due to the differentiation of BMSCs induced by nHAP or nWLKT in both scaffolds, as stem cells will usually become more mature and exhibit growth arrest during osteogenic differentiation [32].

#### 2.3.3. Alkaline Phosphatase (ALP) Activity

Alkaline phosphatase (ALP) is an enzyme found in our body with higher concentrations in bones and the liver. A high level ALP can be observed during the cell maturation and mineralization stage during bone formation. Thus, the elevated ALP levels can be attributed to the production of a mineralized matrix. The ALP activity was shown in Figure S5 (Supplementary Materials), while the normalized ALP activity (to DNA content) is shown in Figure 6C. As can be seen in Figure 6C, the normalized ALP activity on day 0 and 7 was significantly lower compared to those on day 14. Elevation in ALP production thus initiated on day 14, increased further to day 21, and reached a plateau afterwards till day 28. This trend was supported by the early osteogenesis marker nature of ALP [33]. This osteo-induction nature of BMSCs was triggered from nHAP or nWLKT present in the microspheres and thus rationalizes the higher ALP content observed after seven days. Although there was no significant difference in ALP activity between PLGA/nHAP and PLGA/nWLKT at any time points, the latter did show a trend of a higher ALP level compared to the former. More quantitative studies and biochemical tests may be implemented to confirm the difference in relative osteogenic differentiation capability induced by nHAP and nWLKT in PLGA, if there is any.

#### 2.3.4. Immunofluorescent Staining of Type I Collagen (COL I) and Osteocalcin (OCN)

**Figure 7:**
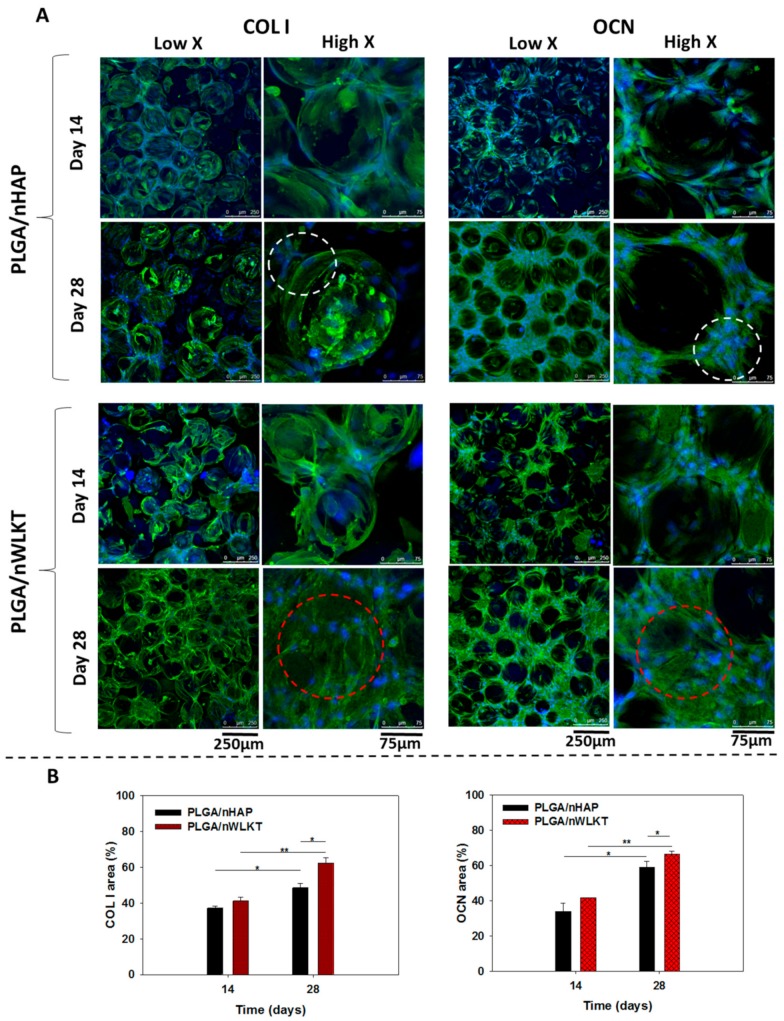
(**A**) Expression of collagen I (COL I) and osteocalcin (OCN) protein by BMSCs when cultured in PLGA/nHAP and PLGA/nWLKT scaffolds on day 14 and 28 by immunofluorescence (IF) staining. The cell nucleus labeled with Hoechst 33,342 is in blue while proteins labeled with FITC-conjugated antibody are in green (bar: low X = 250 µm, high X = 75 µm). (**B**) The semi-quantitative analysis of protein expression developed by an image analysis software (* *p* < 0.05, ** *p* < 0.005).

The osteogenic differentiation potential of PLGA/nHAP and PLGA/nWLKT scaffolds was verified through immunofluorescent (IF) staining of type I collagen (COL I) and osteocalcin (OCN) after observing from confocal microscopy in both low and high magnification (Figure 7A). The scaffolds were tested for the presence of COL I and OCN, which are bone-specific protein markers synthesized by osteoblasts during osteogenic maturation of BMSCs [34]. Presence of proteins were represented by the FITC-green fluorescence while blue is the DAPI-stained nucleus. Production of both COL I and OCN by BMSCs in PLGA/nHAP and PLGA/nWLKT was found to increase with time, with a fluorescence signal from the stained protein distributed around the individual microspheres on day 14 while a more intense fluorescence signal was found to fill the pores between microspheres on day 28. This not only confirmed the more protein production at later stages but also supported the claims that PLGA/nHAP and PLGA/nWLKT induced osteogenic differentiation of BMSCs into osteoblasts in vitro. The empty gap between individual microspheres on day 14 for both scaffolds denoted the absence of cell bridging and thereby endorsed the results observed through SEM, cytoskeletal staining and cell proliferation earlier. Furthermore, the relative intensity of blue colored DAPI stains on day 14 and 28 was not drastically different than for supporting cell growth arrest during differentiation in later cell culture periods. However, there was a trend of higher protein production on day 28 and PLGA/nWLKT appeared to display a relatively higher protein production rate irrespective of when COL I or OCN was used. This could be seen in the higher magnification images where bridging of cells in the pores and hence protein production observed in PLGA/nWLKT was visibly superior (red dashed circles) compared to PLGA/nHAP (white dashed circles).

The qualitative output of protein expression was further substantiated through PAX-it!^TM^ image analysis software quantification, which distinguishes objects by their color, size, or shape [35]. When using this method, the varied color intensity level above a chosen level is compared to the total image area for a quantitative comparison between groups. As shown in Figure 7B, the expression of protein was not significantly different between PLGA/nHAP and PLGA/nWLKT on day 14, although the mean value of PLGA/nWLKT was higher than that of PLGA/nHAP for COL I (48.7 vs. 37.4) and OCN (41.7 vs. 34.0). BMSCs in on both scaffolds produced significantly higher COL I and OCN protein on day 28 compared to day 14. PLGA/nWLKT revealed significantly higher protein expression compared to PLGA/nHAP, which accounted for the cell bridging characteristics observed in Figure 7A. The fact that the respective area percentage of COL I and OCN in PLGA/nWLKT was 1.27 and 1.12 times greater than that in PLGA/nHAP on day 28 may support the use of the former as an bone graft implant in vivo in the bone defective region.

### 2.4. In Vivo Studies

#### 2.4.1. Gross Observation

**Figure 8:**
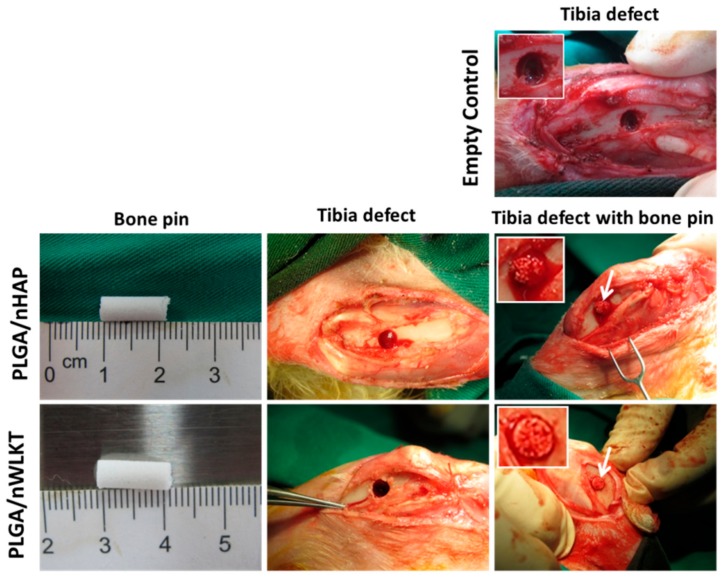
Gross view of the surgical procedure for the implantation of bone pin grafts in rabbit tibia defects. The white arrows denote the implanted bone pine graft. The inserts are enlarged views of the bone defects.

Being an acellular graft implant intended for use as a bone pin or screw, both scaffolds were selected to be implanted in the tibia of rabbits without seeded cells. Due to the complexity of making screw shaped defects, only sintered microsphere scaffold in cylindrical bone pin shape were selected for the in vivo studies. Since both scaffolds were osteoinductive in nature from in vitro studies, it was necessary to evaluate its comparative in vivo bone formation efficiency. Figure 8 shows the gross view images of sterile acellular bone pins prior to implantation, tibia defects created for bone pin grafting, and tibia defects implanted with bone pins. An empty control without bone pin grafting was also shown and included in the experiment. It could be confirmed that both implants (white arrows) were well fitted in the defect cavities through mechanical pressure without leading to any physical deformation, due to their high mechanical strength (Figure 3E,F). The oozing blood observed within the bone pin implanted in tibia from the inserts in Figure 8 confirms that the macroporous structure of the graft is suitable for cell infiltration from surrounding bone tissue. After 12 weeks post-operative period, the rabbits were sacrificed, and the samples were explanted from the animal for gross view observation and histological analysis.

#### 2.4.2. Histological Analysis

**Figure 9:**
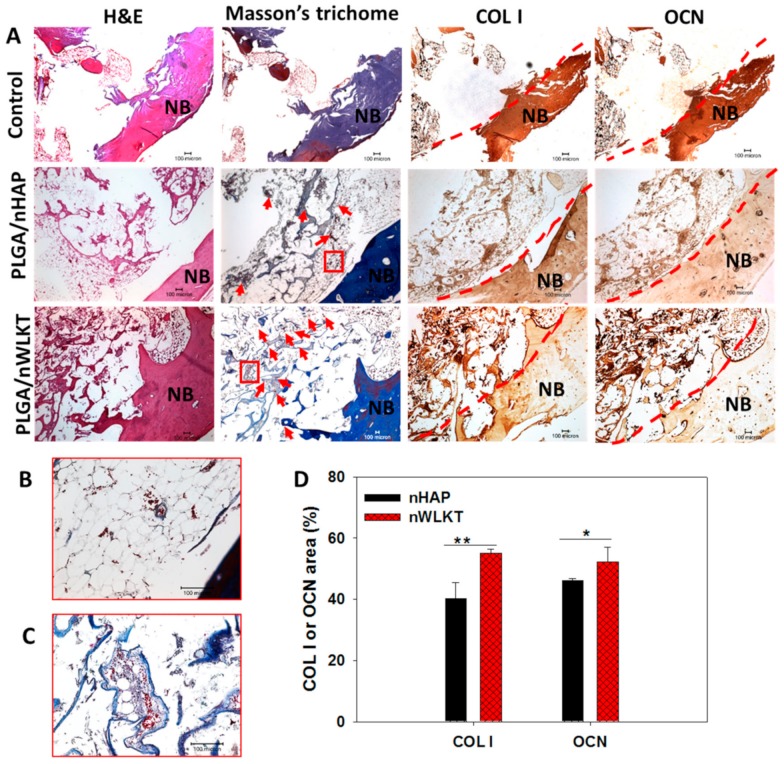
(**A**) The H&E, Masson’s trichrome and immunohistochemical (IHC) staining of COL I and OCN in the rabbit tibia defect repaired with PLGA/nHAP and PLGA/nWLKT microsphere scaffolds (scale bar = 100 µm). The red arrows indicate newly formed blood vessels and NB indicates native bone. The red squares in Masson’s trichrome staining images were further enlarged for PLGA/nHAP (**B**) and PLGA/nWLKT (**C**) to show the newly formed blood vessel. (**D**) The semi-quantitative analysis of protein expression by image analysis from IHC staining (* *p* < 0.05, ** *p* < 0.005).

The regeneration capability of both PLGA/nHAP and PLGA/nWLKT bone pin implants were assessed through histological and immunohistochemical (IHC) staining (Figure 9A). H&E staining showed that relatively fewer cells were found within the defect compared to native bone (NB) in all groups. The narrow rounded appearance of extracellular matrix within the sample should have been due to the infiltration of cells towards the scaffold interior. The irregular empty voids with purple outline morphology confirmed the attachment of infiltrated cells, probably from native bone tissue. This had to be further confirmed with Masson’s trichrome staining, where a deep blue color observed due to the presence of collagen in native bone was evident. Considering the implant area, microsphere circumference was stained light blue for PLGA/nHAP while it was slightly deeper and intense for PLGA/nWLKT. The blue-colored region generally represents the formation of osteoid [36], and thus could be correlated with the initiation of tissue matrix deposition. In comparison with the empty control group, both the PLGA/nHAP and PLGA/nWLKT groups showed higher cellularity from H&E stain and collagen deposition from Masson’s trichrome staining to support the ability of both pin shaped bone grafts to regenerate bone tissue.

The trend seen in both H&E and Masson’s trichrome staining was further confirmed by using COL I and OCN IHC staining. Similar to HE and Masson’s trichrome staining, the empty control group generated very light brown intensities, whereas the microsphere circumference showed brownish staining in the experiment groups. In comparison to the intensities obtained for the PLGA/nHAP scaffold, the PLGA/nWLKT group showed comparatively higher staining density. Previously, WLKT incorporated chitosan scaffolds prepared by freeze drying was shown to have a better rat calvarial bone regeneration ability compared to HAP containing counterparts in vivo [37]. Similarly, PLGA/nWLKT composite scaffolds prepared by salt leaching were found to facilitate bone-specific differentiation in vitro and induce similar or better bone regeneration in a rat calvarial defect model in vivo compared to PLGA/nHAP [17].

The absence of purple colored cell nuclei should be due to the lesser cellular intensity for an acellular scaffold compared to a traditional cellular tissue engineered scaffold. Nonetheless, the results conclude that PLGA/nWLKT and PLGA/nHAP bone pin implants have the ability to allow cellular infiltration into the interiors of the scaffold from surrounding bone. This underlines the potential of surface sintered microsphere scaffolds to be suitable bone grafts in any shapes, without sacrificing the mechanical stability requirement. In addition to this, the Masson’s trichrome staining showed the uneven distribution of red dots that reveals the existence of newly formed blood vessels in the implanted area [38]. It is interesting to note that PLGA/nWLKT shows more pronounced effect to promote blood vessel formation (labeled by red arrows) compared with PLGA/nHAP from the enlarged square areas shown in Figure 9B,C. This property may be correlated with the release of Mg^2+^ ions from WKLT, which can promote angiogenesis [39]. This is consistent with a recent report indicating the angiogenic potential of nWKLT embedded in injectable carrageenan hydrogel in vitro [40], although only minimum angiogenesis effect could be seen here in vivo. To substantiate the preference of PLGA/nWLKT, the IHC staining images were further analyzed semi-quantitatively from image analysis as in IF staining by selecting the area of the scaffold (Figure 9D). The expression of both COL I and OCN in PLGA/nWLKT was found to be significantly higher compared with PLGA/nHAP where the stained area percentage increased by 1.37 fold (COL I) and 1.13 fold (OCN).

In summary, the physico-chemical characterizations and in vitro studies conducted for the PLGA/nHAP and PLGA/nWLKT scaffolds suggest that both scaffolds have comparable properties. However, the in vivo results reveal that PLGA/nWLKT have higher osteogenic potential towards BMSCs compared to PLGA/nHAP bone graft.

## 3. Materials and Methods

### 3.1. Materials

Poly (lactic-co-glycolic acid) (PLGA, intrinsic viscosity = 0.96 dL/g, lactide/glycolide ratio = 85/15) was purchased from Green Square Materials Inc., Taiwan. Poly (vinyl alcohol) (PVA), calcium hydrogen phosphate (Ca_2_HPO_4_·2H_2_O) and cetylpyridinium chloride were obtained from Sigma Aldrich (St Louis, MO, USA). Calcium carbonate (CaCO_3_) was purchased from Scharlau Chemie S.A., Spain. Dichloromethane was obtained from Alfa Aesar (Haverhill, MA, USA). Fetal bovine serum (FBS) was purchased from Gibco (Waltham, MA, USA). Dulbecco’s Modified Eagle’s medium (DMEM) was from Invitrogen (Carlsbad, CA, USA) while ortho-phosphoric acid (85%) was from Merck (Darmstadt, Germany). For nuclear staining, we used 6-diamidino-2-phenylindole dihydrochloride (DAPI) from Life Technologies (Carlsbad, CA, USA). All chemicals were used as received without further purification.

### 3.2. Preparation of Hydroxyapatite Nanoparticles (nHAP)

To prepare HAP nanoparticles from Ca_2_HPO_4_·2H_2_O and CaCO_3_, the chemical precipitation method was used as described before [32]. In this procedure, 25.8 g calcium hydrogen phosphate and 10 g of calcium carbonate were mixed together and added to 1 L of 2.5 M NaOH solution maintained at 75 °C. The mixture was allowed to react for another 1 h, and finally the reaction was terminated by keeping the mixture in an ice bath. Subsequently, the precipitate was washed with double distilled (DD) water, centrifuged, and dried at 70 °C for 24 h to obtain nHAP.

### 3.3. Preparation of Whitlockite Nanoparticles (nWLKT)

The WLKT nanoparticles were prepared by using precipitation of calcium hydroxide magnesium hydroxide with ortho-phosphoric acid, as described previously [16,19,41]. In brief, a conical flask containing 100 mL de-ionized (DI) water was heated up to 80 °C, followed by mixing of 5.48 g of Ca(OH)_2_ and 1.516 g of Mg(OH)_2_ in the solution and was then stirred for 1 h. Subsequently, 100 mL ortho-phosphoric acid (1 M) was added dropwise to the above mixture with continued stirring for another 16 h. The nWLKT particles were separated by centrifugation, washed, and lyophilized for storage.

### 3.4. Preparation of PLGA/nHAP and PLGA/nWLKT Microspheres

The PLGA/nHAP microspheres were prepared based on the emulsion-solvent evaporation technique as described earlier [29]. Briefly, 4 g PLGA was dissolved in 30 mL dichloromethane (DCM) in a vortex shaker and 800 mg of nHAP was added to obtain a solution containing 20% (*w/w*) nHAP. The PLGA solution containing homogenously distributed nHAP nanoparticles were then poured into 1800 mL of 0.5% (*w/v*) PVA solution kept on a magnetic stirrer and allowed to stir at 700 rpm for 18 h for microsphere formation. The microspheres were recovered and washed multiple times with DD water, filtered under vacuum and dried. The PLGA/nWLKT microspheres were prepared using the same method as described before for PLGA/nHAP microsphere. In this case, instead of 800 mg nHAP used for PLGA/nHAP microspheres, 1.2 g of nWLKT was used to prepare 30% (*w/w*) theoretical loading of nWLKT in PLGA/nWLKT. This difference in loading ratio between nHAP and nWLKT was chosen to achieve a similar actual loading percentage of nHAP and nWLKT in the microspheres through pilot experiments. The mixture was then emulsified in 1800 mL of 0.5% (*w/v*) PVA solution kept on a magnetic stirrer and stirred at 700 rpm for 18 h. It was then continued with the same washing and drying steps as before to obtain PLGA/nWLKT microspheres (Figure 1, step 1).

### 3.5. Preparation of PLGA/nHAP and PLGA/nWLKT Microsphere Scaffolds

The PLGA/nHAP and PLGA/nWLKT microspheres were sieved using an array of steel meshes having gradient pore sizes ranging from 88 to 500 µm and microspheres within a size range of 150 to 250 µm was selected for the scaffold fabrication process (Figure 1, step 2). The selected microspheres were filled into a prefabricated cavity shaped stainless steel mold (4 mm height × 4 mm diameter). The microspheres were closely filled inside the cavity by applying pressure using a stainless steel rod that can fit inside the mold cavity. Followed by this, the mold was screw tightened with the mold top and sintered at 83 °C for 90 min. Similarly, the conceptual model of bone pin or screw was made from a pre-fabricated clay based negative mold. A metallic screw was kept inside a square shaped clay, heated at 60 °C, and then the screw was removed by cutting with a surgical knife. The cut pieces from the negative mold were clipped together and microspheres of a selected size range were filled inside through a narrow orifice until the cavity became tight packed. The clay mold was sintered at 86 °C for 90 min to obtain a microsphere based bone pin or screw scaffolds (Figure 1, step 3).

### 3.6. Physico-Chemical Characterization of Nanoparticles, Microspheres and Scaffolds

The morphology of prepared microspheres, microspheres, and scaffolds were characterized using a scanning electron microscope (SEM, S3000N) or a field emission scanning electron microscope (FESEM, SU8010), both of which were from Hitachi, Tokyo, Japan. The elemental composition of nHAP and nWLKT nanoparticles was quantified using energy dispersive X-ray spectroscopy (EDS, Bruker AXS-5030, Billerica, MA, USA). The chemical compositions and the crystallinity of nHAP, nWLKT, and composite microspheres were identified through Fourier-transform infrared spectroscopy (FTIR, RX1, Perkin-Elmer, Waltham, MA, USA) and X-ray diffraction spectroscopy (XRD, D5005, Siemens AG, Munich, Germany), respectively. The thermal behavior of the materials was examined through thermogravimetric analysis (TGA) under nitrogen atmosphere from 25 °C to 700 °C with a platinum pan containing 10 mg of sample in a TA Instruments TGA 2050 thermogravimetric analyzer (New Castle, DE, USA).

The percentage of pores in the microsphere scaffolds (porosity) was measured using the liquid displacement method with a non-solvent of the polymer, ethanol [42]. In brief, pre-weighed microsphere scaffold was placed in a syringe with a known volume of ethanol. Followed by this, the liquid was forced to penetrate into the pores of the scaffold by a series of evacuation/re-pressurization cycles. The porosity was calculated by dividing the volume of ethanol in the scaffold (void volume) with the volume of the whole scaffold (void + solid volume).

The compressive mechanical properties of cylindrical shaped microsphere scaffolds in 4 mm × 4 mm (height × diameter) dimensions were evaluated to confirm their load bearing capacity. The samples were kept on top of the horizontal stainless steel plate and compression mechanical testing was performed using an ElectroForce^®^ 5200 BioDynamic™ Test Instrument (TA Instruments, New Castle, DE, USA) with a 250 N load cell and 0.01 mm/min cross head speed. The mechanical properties of the scaffold were measured in terms of a stress-strain curve, and the Young’s modulus was determined from the initial slope of the stress-strain curve from a 0.01 to 0.05 stain value.

### 3.7. In Vitro Cell Culture Studies

#### 3.7.1. Isolation and Culture of Bone Marrow-Derived Stem Cells (BMSCs)

Rabbit bone marrow-derived mesenchymal stem cells (BMSCs) were isolated from young New Zealand white rabbits as per the standard procedures reported earlier [43]. Animals were anesthetized and the blood was withdrawn from the bone marrow. The anticoagulant (5 mL heparin) was taken in the aspiration needle prior to blood withdrawal and the whole procedures were done according to the rules and regulations of Institutional Animal Care and Use Committee of Chang Gung University (IACUC Approval No.: CGU106-021, 1 July 2017)). Phosphate buffer saline (PBS) was mixed with the blood and the solution was centrifuged at 4 °C. After removing the supernatant, an equal volume of cell culture medium (80% DMEM, 20% FBS, 1% penicillin-streptomycin and 2 μg/mL fibroblast growth factor-2) was added to the concentrated cell solution. Another centrifugation was done, the supernatant was removed and a dark-red solution containing BMSCs was obtained. The cell suspension was uniformly distributed in T-75 flasks with 10 mL of cell culture medium and was incubated at 37 °C. After the removal of non-adherent cells, the BMSCs were further sub-cultured and stored in liquid nitrogen for future studies.

#### 3.7.2. Evaluation of Cell Adhesion and Distribution

The PLGA/nHAP and PLGA/WLKT microsphere scaffolds were sterilized in 75% ethanol under UV for 24 h, rinsed another three times with PBS, and kept in a 24-well cell culture plate (Thermo Fisher Scientific, Carlsbad, CA, USA) containing 1mL cell culture medium (90% DMEM, 10% FBS and 1% antibiotic/antimitotic). After 1 h incubation, we removed the culture medium from each well in the cell culture plate, followed by adding 10 µL of cell suspension containing 1 × 10^5^ BMSCs to each pre-wetted scaffold. The cell-seeded scaffold was placed in a CO_2_ incubator maintained at 37 °C for 4 h to allow cell attachment. The cell/scaffold sample was transferred to another well in the 24-well culture plate, 1 mL of cell culture medium was added, and cell culture analysis was carried out up for to 28 days at 37 °C in a CO_2_ incubator.

The morphology of adhered BMSCs in both microsphere scaffolds was examined through SEM (S-3000N from Hitachi, Tokyo, Japan) while the cytoskeletal arrangements were visualized through a confocal laser scanning microscope (Zeiss LSM 510 Meta, Carl Zeiss AG, Oberkochen, Germany). The samples were first fixed in 2.5% glutaraldehyde solution for 30 min followed through washing with PBS. Alcohol gradient was used to remove water content, followed by critical point drying prior to SEM observation. In cytoskeletal observation, the glutaraldehyde fixed samples were permeabilized with 0.1% Triton X-100 for 10 min at room temperature before staining with red fluorescence-producing phalloidin-tetramethylrhodamine B isothiocyanate (1%, Sigma-Aldrich) and blue fluorescence-producing Hoechst 33,342 (1 µg/mL, Thermo Fisher Scientific).

#### 3.7.3. Cell Viability Analysis

The viability of BMSCs within the microsphere scaffolds was determined through the Live/Dead cell viability assay following standard protocols [44]. The cells were stained with calcein AM/ethidium homodimer in the LIVE/DEAD viability/cytotoxicity kit for mammalian cells (Thermo Fisher Scientific, Waltham, MA, USA). After the staining, the scaffolds were washed with PBS and observed under a confocal laser scanning microscopy (Zeiss LSM 510 Meta).

#### 3.7.4. Cell Proliferation and Differentiation

To evaluate cell proliferation, cell-seeded scaffolds were retrieved at predetermined time points and the DNA content was analyzed using the bis-benzimidazole dye (Hoechst 33258). The DNA was extracted by a digestion buffer containing papain and aliquots of digested solution were mixed with the dye solution and the fluorescence emission was measured. The intracellular alkaline phosphatase (ALP) activity of BMSCs was measured using a SensoLyte pNPP ALP assay kit (AnaSpec, Fremont, CA, USA), using standard protocols. The cell-seeded scaffolds retrieved at specific time points were transferred to the cell lysis solution containing 1 mL of 0.1% Triton X-100 and 5 mM MgCl_2_ for cell lysis and the lysate was centrifuged at 10,000 rpm for 15 min at 4 °C. To measure ALP activity, 50 μL of the lysate supernatant was mixed with 100 μL of 5 mM p-nitrophenyl phosphate (PNPP) solution prepared in 2-amino-2-methyl-1-propanol buffer solution (150 mM). The mixture was allowed to react for 30 min at room temperature in the dark. To terminate the reaction, 50 μL of 0.2 N NaOH was added, followed by measuring the optical density of the solution at 405 nm (OD405) in an enzyme-linked immunosorbent assay (ELISA) reader.

#### 3.7.5. Immunofluorescence (IF) Staining of Bone Marker Proteins

The presence of osteogenic bone markers in the in vitro cultured sample were detected by the immunofluorescence (IF) staining of type I collagen (COL I) and osteocalcin (OCN). The 14 and 28 days BMSCs-seeded samples were collected, washed with PBS, and fixed in 4% formaldehyde for 30 min. Subsequently, the samples were washed with a PBST solution containing PBS and 0.1% Tween 20, for 15 min. The nonspecific proteins present in the sample were blocked by treating the sample with HyBlock 1-min Blocking Buffer^®^ (Goal Bio, Taipei, Taiwan) and washed with PBST again. For COL I, the samples were incubated with antibody for COL I (monoclonal anti-COL I antibody produced in mouse, Novus Biologicals, Littleton, CO, USA) for 8 h at 4 °C, rinsed with PBST for 20 min and incubated in FITC-conjugated goat anti-mouse IgG secondary antibody (Jacksons Laboratories, Bar Harbor, ME, USA) for 1 h. For OCN, the primary antibody used was polyclonal anti-OCN antibody produced in guinea pigs (Cloud-Clone Co., Houston, TX, USA) and the secondary antibody was FITC-conjugated goat anti-guinea pig IgG (Abcam, Cambridge, UK). After we counterstained the cell nucleus with Hoechst 33342, the samples were visualized under a confocal laser scanning microscope (Zeiss LSM 510 Meta) to observe the blue-stained nuclei embedded within the green-stained proteins. For semi-quantitative determination of production of COL I and OCN, the PAX-it!^TM^ image analysis software from PAX-it Inc. (Villa Park, IL, USA) was used. The area percentage of the green fluorescence signal corresponding to COL I or OCN was calculated from six randomly selected fields from microscopic observation and used for protein content quantification.

### 3.8. In Vivo Studies

Eight male New Zealand white rabbits weighing 3 to 4 kg was selected for the study and they were carefully examined for 10 days prior to the experiments and the surgeries were performed under aseptic conditions in accordance with the standard procedures approved by the Institutional Animal Care and Use Committee of Chang Gung University (IACUC Approval No.: CGU106-021, 1 July 2017). Briefly, all rabbits were anesthetized using a mixture of Zoletil 50 (18 mg/kg) and Rompun 20 (1 mg/kg), followed by intramuscularly injecting Atropin (0.3 mg/kg). The tibias of rabbits were shaved. Afterwards, their skin surfaces were disinfected with iodine solution and the tibia part of the leg was carefully exposed via skin incision. After a PBS wash, a 4.2 mm pilot hole was drilled using a round dental bur under profuse saline irrigation. Subsequently, PLGA/nWLKT and PLGA/nHAP pin-shaped scaffolds were inserted into the holes. A control group established by keeping the created hole empty was used for comparison. After the implantation, muscle and skin were sutured separately and the surgical sites were then closed in layers using 4-0 Ethicon sutures. Further, the wounds were sterilized and dressed with gentamicin ointment. All rabbits received intramuscular injections of gentamicin (3 mg/kg) for three days after the surgery and were returned to their normal cages for free movement.

Twelve weeks post-operation, all rabbits were euthanized by lethal doses of pentobarbital and the tibias were dissected out for gross evaluation. The samples from all groups (grafts and control) were fixed in 10% formaldehyde and fixed samples were dehydrated and embedded in paraffin to make 10 µm slice sections on glass slides. Samples were then subject to hematoxylin and eosin (H&E) and Masson’s trichrome staining following standard protocols. Another set of slides were allowed to undergo immunohistochemical (IHC) staining using primary antibodies for COL I (mouse monoclonal anti-collagen I, Novus Biologicals, Littleton, CO, USA) and OCN (mouse monoclonal anti-osteocalcin, Abcam, Cambridge, UK). New bone formation within the implanted PLGA/nHAP and PLGA/nWLKT bone grafts was assessed by recording the images under an inverted optical microscope (Olympus IX-71, Tokyo, Japan).

### 3.9. Statistical Analysis

All quantitative data were expressed as mean ± standard deviation and the statistics used Student *T*-test with a *p* value less than 0.05 considered significant.

## 4. Conclusions

We successfully fabricated macroporous PLGA microsphere based bone grafts containing nHAP (PLGA/nHAP) or nWLKT (PLGA/nWLKT) and compared the properties of each grafts to assess their physico-chemical and osteogenic properties. Most characteristics of both grafts including morphology, mechanical strength, porosity, thermal stability, biocompatibility, and cell viability were found to be similar, suggesting the identical nature of nHAP and nWLKT as a bone graft implant. Nonetheless, judging from COLI and OCN bone marker protein production in vitro and in vivo, PLGA/nWLKT revealed higher osteogenic potential towards BMSCs compared to PLGA/nHAP bone graft. Overall, this study discloses the potential of a relatively less explored nWLKT ceramic nanoparticle as an alternative to nHAP for preparing composite sintered microsphere scaffolds with PLGA for potential bone grafts and tissue engineered implants in pin or screw shapes.

## Supplementary Materials

The following are available online at https://www.mdpi.com/1422-0067/21/2/528/s1. Figure S1. The SEM images of PLGA microspheres at different magnifications. Figure S2. The optical microscopic images of PLGA microspheres obtained at different PLGA concentrations for the optimization of PLGA concentration. Figure S3. The TGA results for the assay conducted for microsphere scaffold containing two different concentrations of nWLKT for the optimization of nWLKT loading. Figure S4. The SEM images of low and high magnification of PLGA/nHAP microsphere scaffold (A and B) and PLGA/nWLKT microsphere scaffold (C and D). Figure S5. The ALP activity of BMSCs in PLGA/nHAP and PLGA/nWLKT microsphere scaffolds.
