# Supplementary material for: Preparation and Characterization of Surface Heat Sintered Nanohydroxyapatite and Nanowhitlockite Embedded Poly (Lactic-co-glycolic Acid) Microsphere Bone Graft Scaffolds: In Vitro and in Vivo Studies
Source: Int J Mol Sci. 2020 Jan 14;21(2):528. doi: 10.3390/ijms21020528 (PMC7013730; doi:10.3390/ijms21020528)
Supplement: Supplementary file 1 [file ijms-21-00528-s001.pdf]

Supplementary Material

Preparation and Characterization of Heat Sintered Nanohydroxyapatite and Nanowhitlockite Embedded Poly(lactic-co-glycolic acid) Microsphere Bone Graft Scaffolds: In Vitro and In Vivo Studies

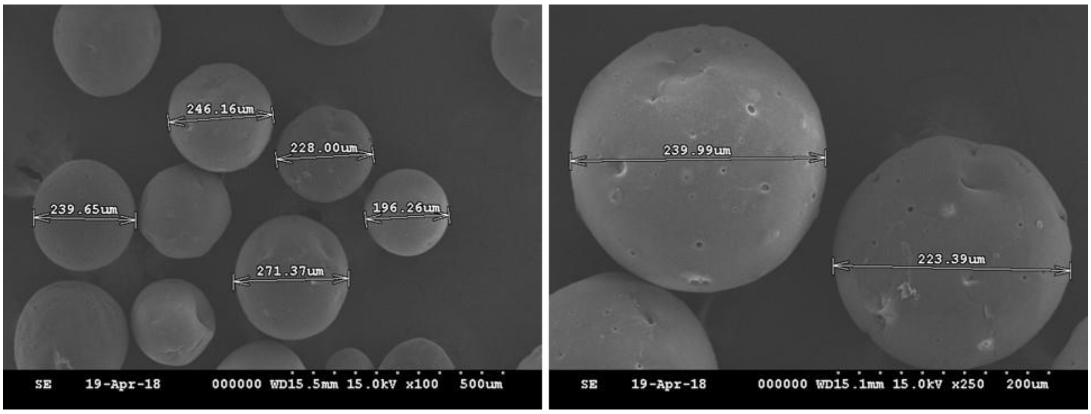

Figure S1. The SEM images of PLGA microspheres at different magnifications.

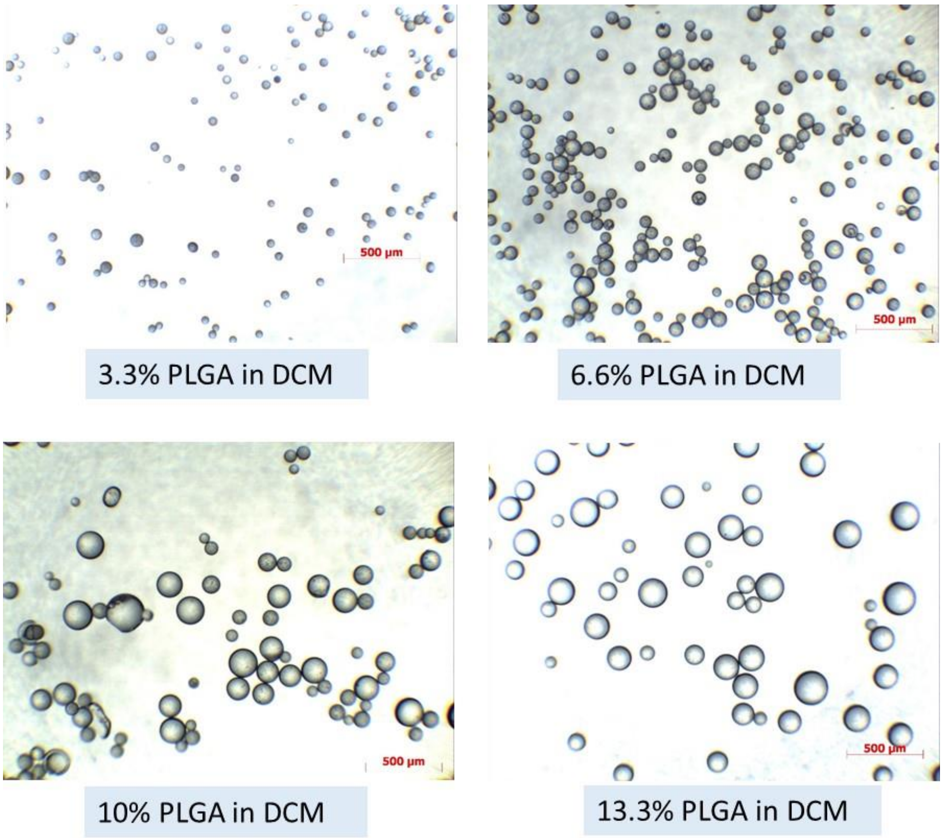

Figure S2. The optical microscopic images of PLGA microspheres obtained at different PLGA concentrations for the optimization of PLGA concentration.

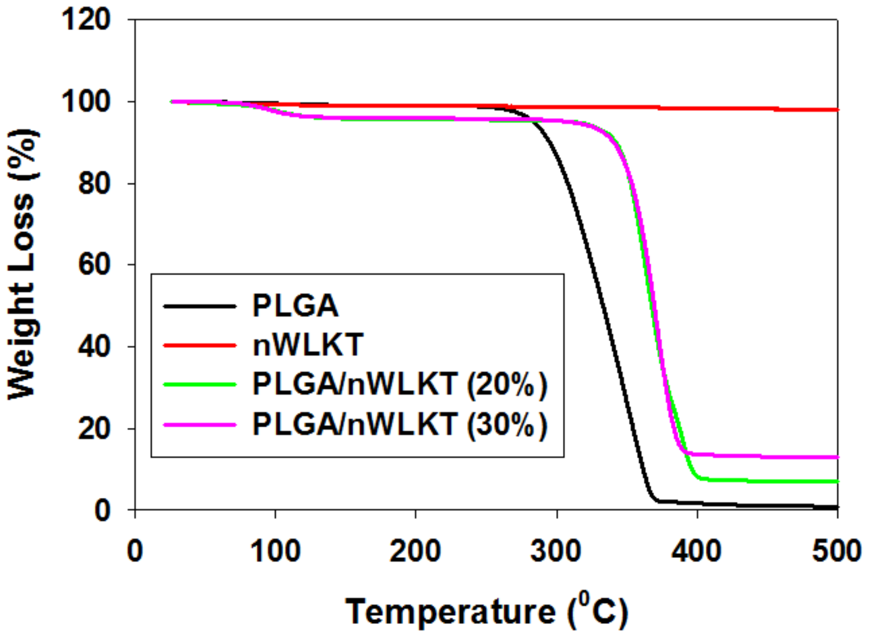

Figure S3. The TGA results for the assay conducted for microsphere scaffold containing two different concentrations of nWLKT for the optimization of nWLKT loading.

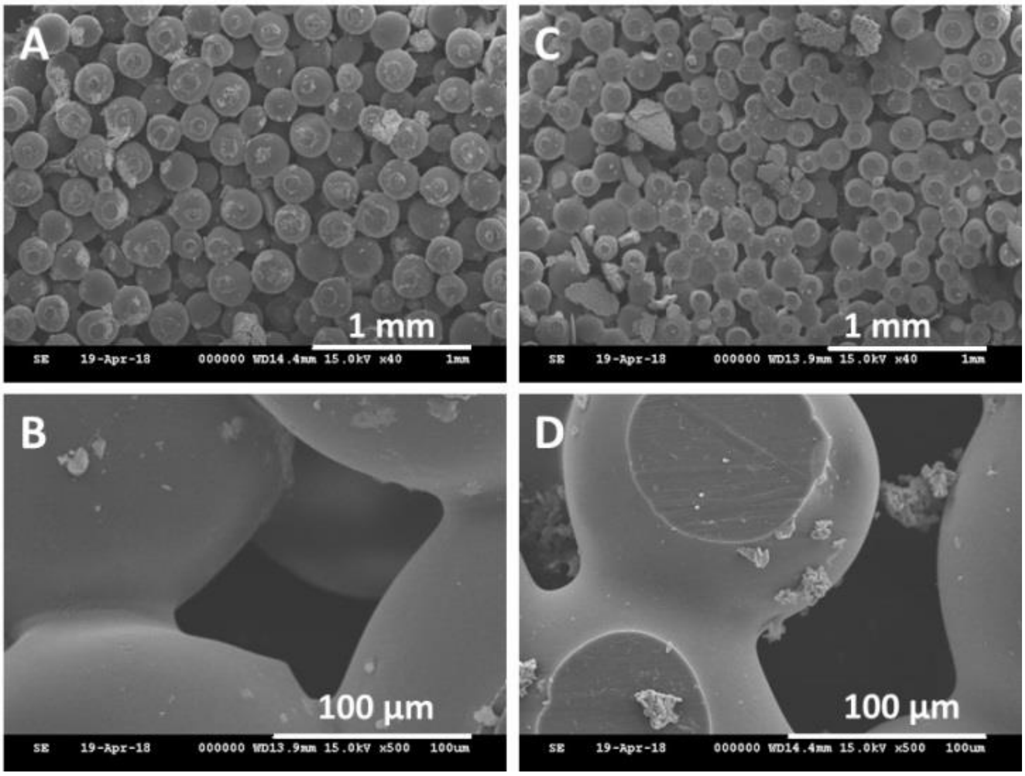

Figure S4. The SEM images of low and high magnification of PLGA/nHAP microsphere scaffold (**A and B**) and PLGA/nWLKT microsphere scaffold (**C and D**).

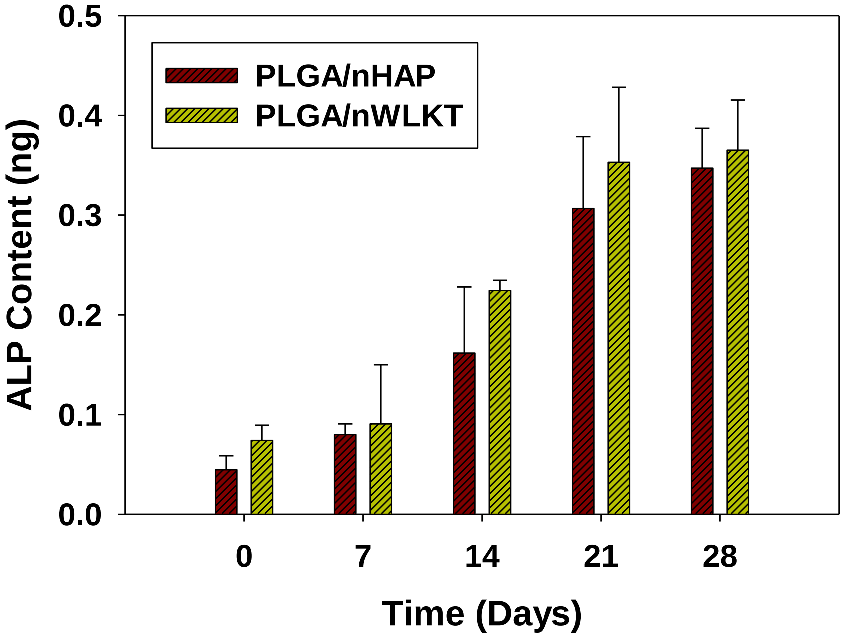

Figure S5. The ALP activity of BMSCs in PLGA/nHAP and PLGA/nWLKT microsphere scaffolds.
